# The highly conserved chromosomal periodicity of transcriptomes and the correlation of its amplitude with the growth rate in *Escherichia coli*

**DOI:** 10.1093/dnares/dsaa018

**Published:** 2020-08-31

**Authors:** Motoki Nagai, Masaomi Kurokawa, Bei-Wen Ying

**Affiliations:** Graduate School of Life and Environmental Sciences, University of Tsukuba, Tsukuba, Ibaraki 305-8572, Japan

**Keywords:** chromosomal periodicity, transcriptome, growth rate, population fitness

## Abstract

The growth rate, representing the fitness of a bacterial population, is determined by the transcriptome. Chromosomal periodicity, which is known as the periodic spatial pattern of a preferred chromosomal distance in microbial genomes, is a representative overall feature of the transcriptome; however, whether and how it is associated with the bacterial growth rate are unknown. To address these questions, we analysed a total of 213 transcriptomes of multiple *Escherichia coli* strains growing in an assortment of culture conditions varying in terms of temperature, nutrition level and osmotic pressure. Intriguingly, Fourier transform analyses of the transcriptome identified a common chromosomal periodicity of transcriptomes, which was independent of the variation in genomes and environments. In addition, fitting of the data to a theoretical model, we found that the amplitudes of the periodic transcriptomes were significantly correlated with the growth rates. These results indicated that the amplitude of periodic transcriptomes is a parameter representing the global pattern of gene expression in correlation with the bacterial growth rate. Thus, our study provides a novel parameter for evaluating the adaptiveness of a growing bacterial population and quantitatively predicting the growth dynamics according to the global expression pattern.

## 1. Introduction

The growth rate in the exponentially growing phase is the most important parameter representing both genetic and environmental influences on bacterial growth dynamics. Predicting the growth rate of a growing bacterial population according to the intrinsic status and/or extrinsic conditions is highly desirable. To date, extensive studies involving the systematic quantitative investigation of bacterial growth have been performed. By using systematic manipulation or engineering, e.g. single-gene knockout^1^ and genome reduction,^2^^,^^3^ the contributions of single genes and large genomic fragments to bacterial growth were quantitatively evaluated.^4–6^ The correlation identified between the genome size and the growth rate strongly suggests that population fitness is linked to genome-wide features (e.g. transcriptome, mutation rate) *in vivo*.^5^^,^^7^

The transcriptome, which illustrates a global view of the transcriptional abundance of all the genes in the genome, is reorganized constantly in response to genomic and environmental perturbations.^8–10^ As the transcriptome is known to be associated with population fitness,^11–13^ the contribution of the transcriptome to population fitness is of great interest. Our previous studies revealed the coordination of gene expression with the growth rate^13^ and the linkage between transcriptome reorganization and increases in fitness in adaptation and evolution.^14^^,^^15^ These findings indicated that the transcriptome, rather than the specific regulation of limited gene groups, increased population fitness. However, whether and how the transcriptome is linked to population fitness remains unknown.

A single parameter representing the transcriptome is critical for determining the linkage if it exists. Previous studies demonstrated that the power law (Zipf’s rule) was a universal principle governing the transcriptome in living organisms;^16^^,^^17^ however, we failed to find the linkage between this law and the growth rate.^18^ As an alternative global feature representing the transcriptome, the chromosomal periodicity of transcriptome has been proposed,^19^^,^^20^ which is determined using the Fourier transform, a mathematical method used to estimate the periodic patterns in an entire data set according to the sinusoidal wave.^21^ Applying the Fourier transform to the transcriptome data set could estimate the periodic patterns of gene expression levels across the entire chromosome. Computational analyses identified some particular periods associated with bacterial transcriptomes^22^ of distinctive spatial patterns across the chromsomes.^19^^,^^20^ These chromosomal periodic patterns were proposed to be contributed by the chromosomal topology, which related to either the chromosomal domain structure or the specific DNA contents.^23–25^ These findings of chromosomal periodicity were used to obtain static snapshots of the transcriptome, but whether and how the chromosomal periodicity of the transcriptome is linked to the growth rate are unknown.

In the present study, a total of 213 growth profile-associated transcriptomes were analysed that represent an assortment of *E. coli* strains growing under or responding to various environments. This study seeks to determine whether the chromosomal periodicity of the transcriptome is robust or variable in response to environmental and genetic perturbation and whether and how chromosomal periodicity is coordinated with bacterial growth.

## 2. Materials and methods

### 2.1. *Escherichia coli* strains and growth conditions

Three types of *E. coli* genomes were included in the transcriptome analyses: the full-length genomes of MG1655 and DH1 and the reduced genome of MDS42.^2^ A number of genetically engineered strains were comprised of genomes of types DH1^14,26^ and MDS42,^13^ which led to an assortment of genetic backgrounds. The growth media were all based on the minimal medium M63;^27^ if required, the medium was supplemented with nutritional ingredients, such as, leucine and histidine, to compensate for the lost gene function resulting from genetic engineering. In addition, the growth temperature was varied from 37°C to 45°C. The *E. coli* cells either in the exponential growth phase or at the stress response phase were subjected to the analyses. In brief, a total of 72 combinations of the genetic and conditional variations were acquired, which were summarized in Supplementary Table S1. The details of the genetic manipulation and the experimental conditions can be found in previous reports.^13–15^^,^^18^^,^^26^^,^^28^^,^^29^

### 2.2. Transcriptome data sets

The transcriptome data sets used in the present study were obtained from the microarray raw data assigned with the GEO access numbers of GSE33212, GSE49296, GSE55719, GSE52770 and GSE61749 by using the customized platform EcFS.^30^ The finite hybridization model^31^ was applied to determine the gene expression levels, which were calculated as the log-scale mRNA concentrations (pM). Data filtering, normalization and averaging of the biological replicates for the subsequent transcriptome analyses were described previously.^13^^,^^28^ The resulting transcriptome data sets were associated with the growth profiles (e.g. growth rate and growth phase), genomic backgrounds and environmental conditions. The details were previously described in the corresponding studies.^13–15^^,^^26^^,^^28^ A total of 213 transcriptome data sets, comprising 72 combinations that varied in terms of the genomic background and environmental conditions as described above (Supplementary Table S1), were included in the analyses. For correlation analysis, we only used the transcriptome data sets that were obtained at the exponential growth phase and thus linked to precise growth rates.

### 2.3. Computational analyses

All computational analyses were performed with R.^32^ The gene expression levels on a logarithmic scale were used for the analyses as described previously.^18^^,^^33^ The transcriptome data sets obtained at the exponential growth phase (Supplementary Table S1) were subjected to correlation analysis, that is, the correlations with the growth rate (**r**) and the periodic parameters (**a**, **b** and **c**). The parameters of **a**, **b**, and **c** represented the amplitude of the period, the phase of the period and the mean transcriptional level, respectively. The statistical significance of the Pearson correlation coefficients was evaluated by the *t*-test. The *Z*-score was used for the standardization of the MG1655 transcriptomes obtained from the present data sets and the GyrA Chip-seq data for MG1655 obtained from another study.^20^ The *Z*-scores of both the gene expression and the GyrA binding were calculated and averaged for 100-kb bins with a sliding distance of 1 kb for the correlation analysis and the Fourier transform. To compare the chromosomal periodicities of all transcriptomes, the genome position was normalized in the range of 0∼1, simply by dividing the respective length of genome sequence, because the transcriptomes were from different *E. coli* strains of varied genome sizes. In addition, the start points of the genomes were according to the genomic sequences deposited in DDBJ and were identical for all transcriptomes.

### 2.4. Evaluation of chromosomal periodicity

A standard Fourier transform was employed to determine the chromosomal periodicity of the transcriptomes and the GyrA binding activity by using the periodogram function in R. All 213 transcriptomes were subjected to the Fourier transform, in which the expression data for 4,393, 3,760 and 4,377 genes in the *E. coli* strains with the genomic backgrounds of MG1655, MDS42 and DH1 were used, respectively (Supplementary Table S2). The CDS information for MG1655, MDS42 and DH1 were obtained from the DDBJ databank under the accession IDs U00096, AP012306 and AP012030, respectively. The sizes of the genomes used for the Fourier transform were 4,642, 3,976 and 4,622 kb for MG1655, MDS42 and DH1, respectively. The chromosomal periodicities of both the transcriptomes and the GyrA binding activity were evaluated with a sliding distance of 1 kb and are shown in 100-kb bins. The approximate curves of the periodicity were calculated using the highest peak of the periodogram and were fitted by minimizing the square error of the approximate curve and the series of expression values. The statistical significance of the periodicity was assessed with Fisher’s g test,^21^ which was performed using the GeneCycle package in R.

## 3. Results and discussion

### 3.1. The common chromosomal periodicity of the transcriptome

**Figure 1:**
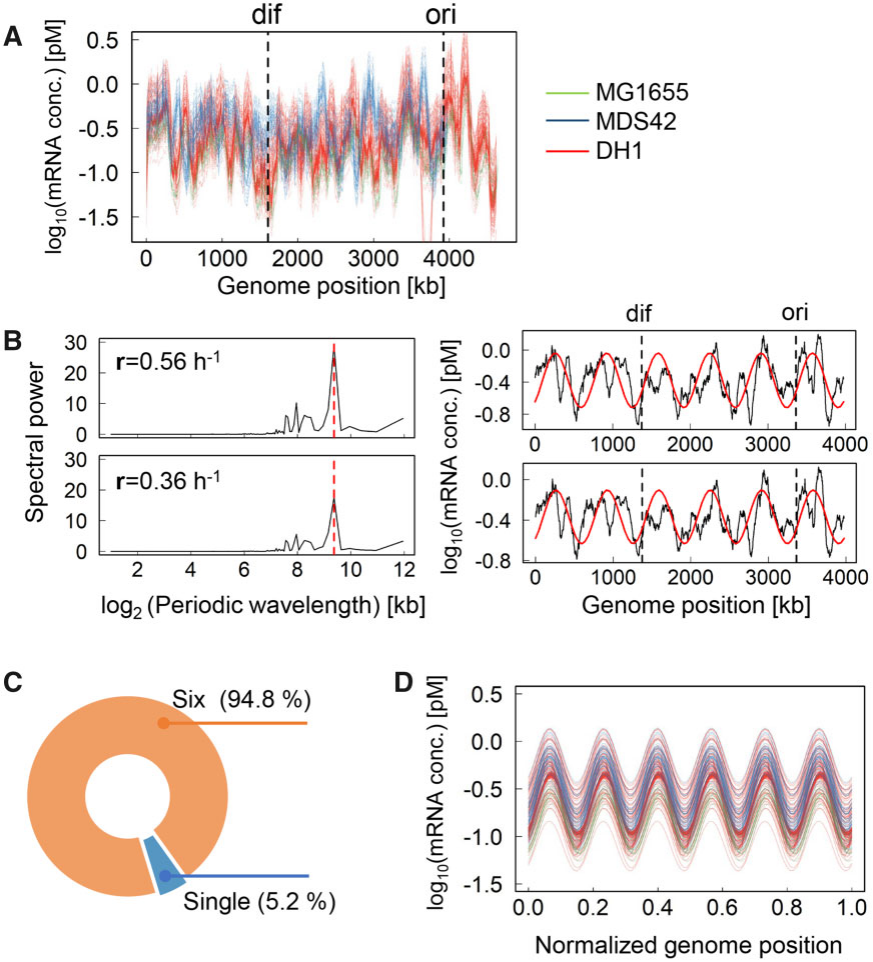
Chromosomal periodicity of the transcriptome. (A) All transcriptomes used in the present study. The transcriptional levels of every 1-kb sliding window and 100-kb smoothing are shown. The colour variation indicates the transcriptomes of individual strains. *Ori* and *dif* are indicated by the broken lines in black. (B) Periodograms of the transcriptomes. The upper and bottom panels indicate the transcriptomes of the *E. coli* cells (MDS42) grown at 40.0°C and 41.5°C, respectively. The growth rates, **r**, are indicated in the insets. The left and right panels represent the distributions of the Fourier-transformed periodic wavelengths on a logarithmic scale and the estimated chromosomal periodicity of the transcriptome, respectively. The broken lines and solid curves in red indicate the highest power spectra (the max-peak) estimated by the Fourier transform and the corresponding fitted period of the transcriptome, respectively. The transcriptional levels for every 1-kb sliding window and 100-kb smoothing are shown. *Ori* and *dif* are indicated by the broken lines in black. (C) Distribution of the periods corresponding to the max-peak. Orange and blue indicate the ratios of the six periods and the single period among the 213 total transcriptomes, respectively. (D) Overlapping periods of the transcriptomes. The chromosomal periodicity of 202 transcriptomes showing six periods (orange in C) were plotted together. The colour variation corresponds to that shown in (A).

To investigate whether the growth conditions and the genomic background influenced the chromosomal periodicity of the transcriptome, a total of 213 *E. coli* transcriptome data sets, which were associated with the growth profiles and were acquired with the same microarray platform, were used in the present study. The growth conditions were varied in terms of temperature, nutrition level and osmotic pressure, and there was a large variation in the genomic backgrounds (Supplementary Table S1, as described in Materials and methods). Fluctuations in gene expression across the entire genome were confirmed (Fig. 1A), and chromosomal periodicity was evaluated with the Fourier transform.

Intriguingly, the analysis results showed the highly conserved chromosomal periodicity of the transcriptomes, which was independent of the growth conditions and the genomic backgrounds. For instance, the most significant spectral powers (i.e. the max peak of the predicted wavelength) identified in two transcriptomes associated with different growth rates (**r**) were exactly the same (Fig. 1B, left panels). Consequently, this resulted in an identical chromosomal periodicity (Fig. 1B, right panels), although the two transcriptomes represented the *E. coli* cells growing at different temperatures. Overall, 202 out of 213 transcriptomes presented a common chromosomal periodicity of six periods (Fig. 1C) as the highest priority in the Fourier transform. Of note, all 11 exceptions showed a chromosomal periodicity of six periods as the second priority and that of a single period as the first priority (Supplementary Fig. S1). Neither specific genetic background nor particular growth condition was detected in these exceptions. As the statistical significance of the chromosomal periodicity was further proven by Fisher’s g test for all transcriptomes (Supplementary Fig. S2), the determination of the common chromosomal periodicity of transcriptomes, which consisted of six periods, was highly reliable. This result agreed with those of previous studies reporting the periodic transcriptomes of six or more periods either in wild-type *E. coli* strains or under regular growth conditions.^20^^,^^22^^,^^28^

In addition, the chromosomal periodicity of the transcriptomes was somehow synchronized. Despite the large variation in both the genomic backgrounds of the *E. coli* strains and the environmental conditions of the population growth, the six periods of a total of 202 transcriptomes almost overlapped (Fig. 1D), which indicated the similarity in the phase of periodic transcriptome. Such similar directional changes in gene expression among the genomic positions (i.e. overlapped phase of periods) further demonstrated the universality of the chromosomal periodicity of the transcriptomes irrespective of genetic and environmental disturbances. This was the first finding that revealed that neither the number of the periods nor the wavelength was linked to bacterial growth condition.

### 3.2. Correlation between the growth rate and the amplitude of the periodic transcriptome

Whether there was any parameter representing a feature of the chromosomal periodicity of transcriptomes, which is linked to bacterial growth, was further investigated. The gene expression level, Exp(*x*), was related to the genome position (*x*) of the corresponding gene. The parameters affecting the chromosomal periodicity of the transcriptome were theoretically defined in the following formula (Eq. 1).

(1) $$\mathrm{Exp}\;(x)=a\times \sin\left(\frac{x+b}{T}\times 2\pi \right)+c$$

**Figure 2:**
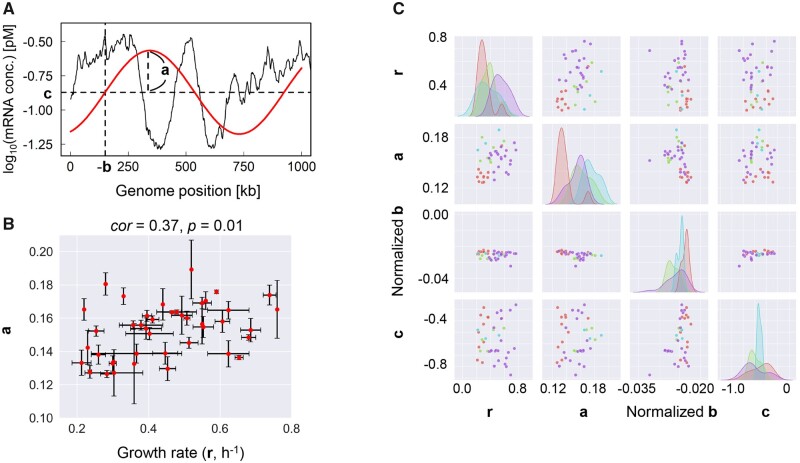
Correlation between the chromosomal periodicity and the growth rate. (A) Illustration of the parameters defined for the periodic transcriptome. The parameters (**a**, **b** and **c**) used in Equation (1) are indicated. Black and red lines represent the transcriptome and the fitted period, respectively. (B) Scatter plot of the amplitude of the six periods and the growth rate. A total of 42 different combinations of transcriptomes are indicated as the red dots. The standard errors of the biological replicates are indicated. The correlation coefficient and its significance are indicated. (C) Relationships among the parameters defined for the chromosomal periodicity and the growth rate. The relationship between any two of **a**, **b**, **c** and **r** is shown in matrix form. Pink, blue, green and purple represent the environmental variations in temperature, osmotic pressure, and nutritional level and regular conditions, respectively. The cells of **r**/**r**, **a**/**a**, **b**/**b** and **c**/**c** show the distributions of **r**, **a**, **b** and **c**, respectively. The vertical axes stand for the frequencies of the corresponding parameters in varied scales.

Here, the parameters **a**, **b**, and **c** represented the amplitude of the period, the phase of the period (i.e. the genomic position of the period initiation), and the mean transcriptional level, respectively (Fig. 2A). The estimation of the three parameters was performed by minimizing the square error in the curve fitting. The constant *T* was the wavelength of the period of the highest spectral power estimated by the Fourier transform. The transcriptomes (42 different combinations), which represented the exponential growth phase and were associated with highly precise growth rates (**r**), were subjected to theoretical fitting with Equation (1).

The theoretical fitting successfully identified a significant correlation between the growth rate and the amplitude of the periodic transcriptome. The values of **a**, **b** and **c** were calculated by curve fitting with Equation (1), and subsequently averaged among the biological repeats. Note that parameter **b** was further normalized because of the variation in the genome length. The parameter **a** was positively correlated with the growth rate (Fig. 2B), whereas such a correlation was not detected for the parameters **b** and **c** (Supplementary Fig. S3). The analysis clearly determined a simple correlation between the growth rate and the amplitude of the period, although the transcriptomes/combinations largely differed in terms of the genotypes and environments (Supplementary Table S1). This correlation strongly suggested that population fitness was associated with the magnitude of differential transcription along the chromosome. This finding provided an intriguing linkage between the bacterial growth and the significance of transcriptomic changes.

Investigations of the contributions of the genomic background and the environmental conditions failed to observe any significant relationship with the growth rate. According to previous reports,^13–15^^,^^18^^,^^26^ four types of environmental variations were roughly categorized as regular (no stress) conditions and conditions with changes in temperature, nutrition level and osmotic pressure. The distributions of the four parameters representing the growth and the periodicity of the transcriptome were largely dissimilar among the four categories (Fig. 2C), which reflected the properties of the data sets. No environment-dependent feature or correlation among the parameters **a**, **b**, and **c** was found (Fig. 2C). Additionally, genetic engineering might affect the phase of the period (i.e. normalized **b**), as a difference was detected between the wild-type genome and the other genomes (Supplementary Fig. S4). The genetic reconstruction possibly changed the genomic position of the period initiation, although more data sets were required to support this assumption.

### 3.3. Mechanisms of the conserved chromosomal periodicity of transcriptomes

To understand the universality of the periodicity of transcriptomes, a simple assumption was made that the essential genes determined the six periods. A total of 302 genes were experimentally determined to crucial for *E. coli* in common (https://shigen.nig.ac.jp/ecoli/pec/).^34^ Such essentiality might have determined the chromosomal periodicity. However, neither deleting the essential genes from the transcriptome nor substituting the true expression values with zero altered the common periodicity of the transcriptome (Supplementary Fig. S5). This result indicated that the periodicity of the transcriptome was not simply due to the genomic localization of the essential genes.

**Figure 3:**
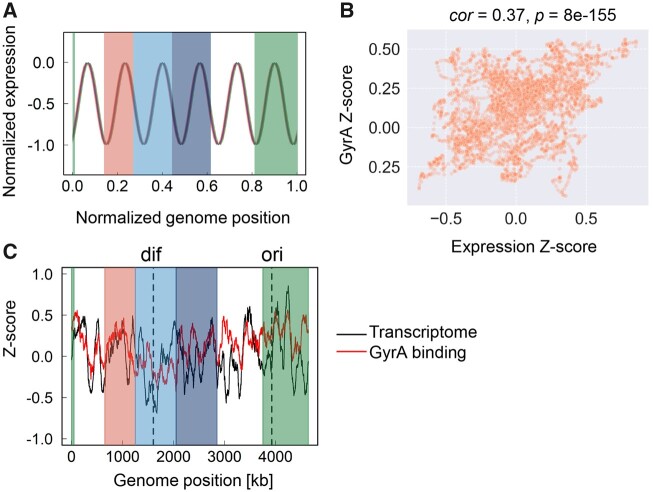
Comparison of the common periodicity of the transcriptomes to chromosome structures. (A) Relationship between the chromosomal macrodomains and the periodic transcriptomes. The normalized periodic transcriptomes are shown. Four macrodomain regions and two non-structural regions are shown in solid colour and as transparent, respectively. The macrodomains of the Ori, Right, Ter and Left regions are shown in green, red, light and dark blue, respectively. (B) Scatter plot of the transcriptome versus GyrA binding activity in MG1655. Standardization of both the mean expression levels and the GyrA Chip-seq data were performed by determining the *Z*-score. The correlation coefficients and the statistical significance are indicated. (C) Comparison of the transcriptome and the GyrA binding activity of the wild-type genome MG1655. Red and black curves indicate GyrA binding activity and the transcriptome, respectively. Both were calculated using a 1-kb sliding window and are shown according to the 100-kb moving average. *Ori* and *dif* are indicated by the broken lines in black.

As the domain structure of chromosome might contribute to transcriptional activity,^33^^,^^35–37^ whether the common chromosomal periodicity of the transcriptome was attributed to the chromosomal organization was determined. The macrodomain model was proposed for the *E. coli* chromosome, with four structured domains and two non-structural regions.^38–41^ The normalized periodicity of the transcriptomes showed that the six periods were roughly positioned within the six domain regions of the *E. coli* chromosome (Fig. 3A), which was consistent with previous findings that showed the similar six periods.^20^^,^^22^^,^^28^ As the six periods of transcriptome linked to the six domain regions of the chromosome, the highly overlapping phases of the periodic transcriptome (Fig. 3A) suggested that the chromosomal macrodomain structure remained conserved, regardless of the genomic and environmental disturbances.

Moreover, the DNA topology-related molecular mechanisms may play a role in determining the chromosomal periodicity of the transcriptome. Bacterial chromosomal structures are highly dynamic and compacted in association with nucleoid-associated proteins (NAPs). Previous studies indicated that the chromosomal supercoiling of ∼10-kb domains^20^^,^^23^ was potentially attributed to the chromosomal localization of NAPs, e.g. H-NS,^42^ and the chromosomal supercoiling of 600∼800-kb domains might be triggered by DNA gyrase.^20^ As the common period identified in the present study was ∼700 kb, which was similar to the domain size triggered by DNA gyrase, whether GyrA, the functional subunit of DNA gyrase, participated in the chromosomal periodicity of transcriptome was analysed. A weak but statistically significant correlation was verified between the transcriptome of the wild-type strain MG1655 in the present study and the abundance of chromosomally bound GyrA in the exponentially growing MG1655 in a previous report^20^ (Fig. 3B). Such a correlation seemed to be common in all transcriptomes (Supplementary Fig. S6) if the properties of the binding of GyrA to the genome remained unchanged. So far, it was unknown whether the variation in either the genomes or the growth phases caused any changes in the GyrA binding activities. This weak correlation between gene expression and binding activity suggested somehow the similarity of the chromosomal periodicity of GyrA binding and that of the transcriptome. Although the six periods were not the first priority for GyrA binding (Supplementary Fig. S7), the chromosomal periodicities of GyrA binding activity and the transcriptome in MG1655 were somehow coordinated (Fig. 3C). As the chromosomal macrodomain structure was reported to be relaxed in the stationary phase,^42^ the high-throughput experiments directly linking the growth phase, genome, transcriptome and the GyrA binding activity are required for the precise verification of the correlations. Additionally, the imperfect positioning (coordination) of the *ori*/*dif* domains to the corresponding periods of transcriptomes might be resulted from either the divergence of the experimental and computational approaches or the difference of genome size.

### 3.4. Linking the periodicity of the transcriptome to population fitness

**Figure 4:**
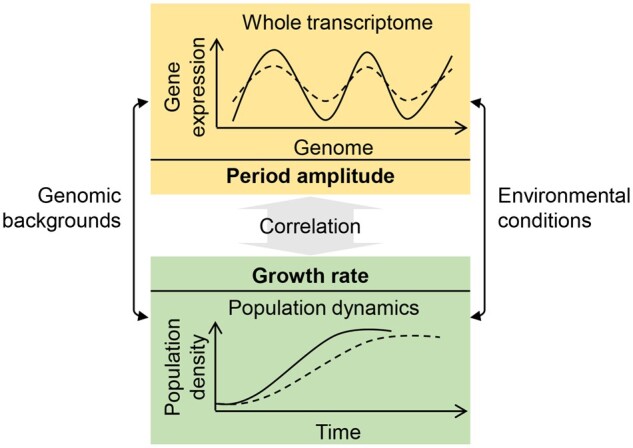
Scheme of the correlation between the growth rate and the chromosomal periodicity of the transcriptome. Shadowed boxes indicate the profiles of the transcriptome and the corresponding population dynamics. The broken and solid curves in the upper box indicate the small and large amplitudes of the periodic transcriptomes, respectively, and those in the bottom box indicate slow and fast growth, respectively.

The present study successfully found a direct linkage between population dynamics and the transcriptome (Fig. 4). The transcriptome is influenced by both the genomic background and the environmental conditions; consequently, it determines bacterial growth. Previous studies of transcriptomes successfully classified the genes into diverse categories that functioned either specifically in response to environmental changes or generally in relation to the growth rate.^11–13^ These achievements linked the individual gene function and/or gene expression to the growth. However, whether there was any quantitative relationship directly linking the transcriptome to the growth remained unaddressed.

The present study first identified a single parameter (i.e. the parameter of **a**) that represented well the overall feature of the transcriptome. That is, the amplitude of the chromosomal periodicity of the transcriptome represents the magnitude of the changes in gene expression across the genome. Moreover, fast growth was linked to a large amplitude of the periodic transcriptome (Fig. 4, solid curves). The correlation between the growth rates and the amplitudes of periodic transcriptomes was independent of the environmental conditions and the genomic backgrounds. This novel finding was a breakthrough for understanding how the transcriptome determined population fitness because it was the first demonstration that the magnitude of chromosomal differentiation of gene expression was correlated with the growth rate. The additional analysis failed to find the correlation between the genomic sequence (the GC-content) and the chromosomal periodicity of transcriptomes (Supplementary Fig. S8). It is currently unknown whether the genome replication links to the periodic transcriptome, what the benefits are for the *E. coli* to possess the conserved chromosomal periodicity of transcriptomes, and whether the six-period is an evolutionary consequence. These are highly intriguing questions required to be addressed in the future.

The growth rate was the only global parameter representing the adaptiveness of a growing bacterial population. The amplitude of the periodic transcriptome could be considered as an alternative to the growth rate, which is a global parameter for evaluating the population fitness. Further studies are required to investigate the mechanisms for such a periodicity and its physiological role. In addition to network reconstruction,^43^ the assessment of the global pattern of the transcriptome might be applied for predicting population fitness, which would be beneficial for industrial applications to microbial production of various chemicals and biological materials and the fundamental investigation of living principles.

## Supplementary Material

dsaa018_Supplementary_Data
