# Supplementary material for: The highly conserved chromosomal periodicity of transcriptomes and the correlation of its amplitude with the growth rate in Escherichia coli
Source: DNA Res. 2020 Aug 31;27(3):dsaa018. doi: 10.1093/dnares/dsaa018 (PMC7508348; doi:10.1093/dnares/dsaa018)
Supplement: dsaa018_Supplementary_Data [file dsaa018_supplementary_data.zip › Revised_Supplemental_200803-.docx]

**
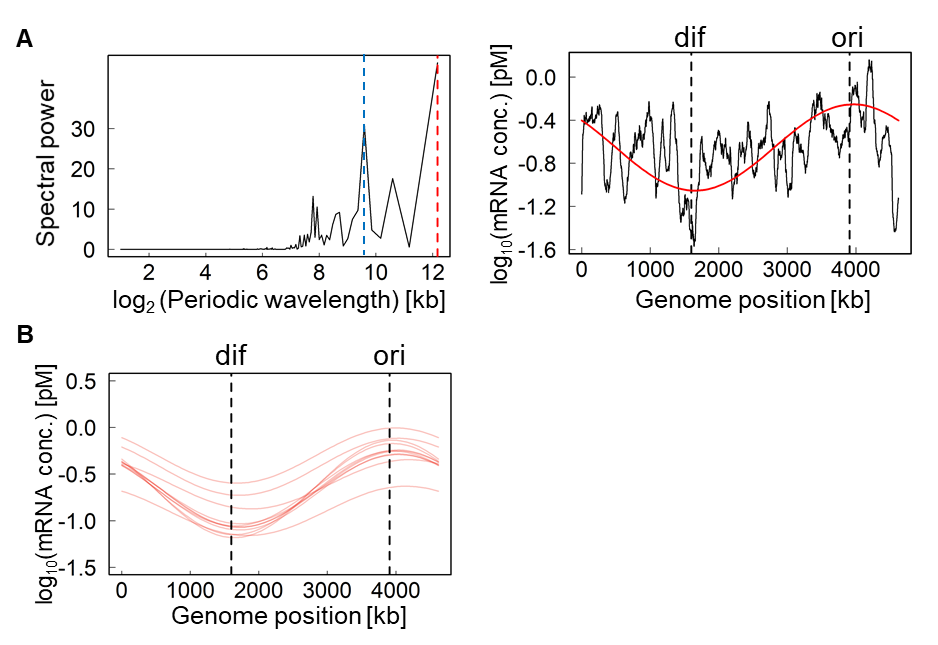
**

**Figure S1 Chromosomal periodicities of a total of 11 exceptional transcriptomes**. **A.** An example of the periodograms of the exceptional transcriptome. The left and right panels represent the distributions of the Fourier-transformed periodic wavelengths at a logarithmic scale and the estimated chromosomal periodicity of the transcriptome, respectively. The broken line and solid curve in red indicate the highest spectral power (the max-peak) estimated by the Fourier transform and the corresponding fitted period of the transcriptome. The broken line in blue indicates the spectral power of second priopority, which represents a chromosomal periodicty of six periods. *Ori* and *dif* are indicated by the broken lines in black. **B.** Overlapped single periods of 11 exceptional transcriptomes. The chromosomal periodicity of a total of 11 exceptional transcriptomes all showed a single period. These exceptions shared the same genome of DH1, whereas, the growth conditions were varied (*i.e.*, three of regular condition, two of heat stress condition and six of starved condition). *Ori* and *dif* are indicated by the broken lines in black.

**
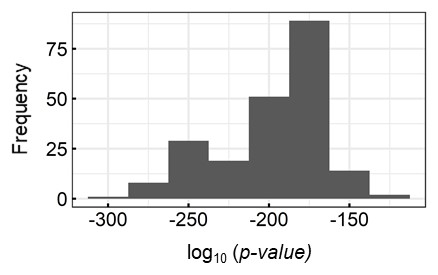
**

**Figure S2 Histogram of the significance of the chromosomal periodicity.** Fisher’s g-test was performed to evaluate the statistical significance of the common six periods of the transcriptomes. Frequency indicates the number of the transcriptomes (Y-axis). The *p*-values of the Fisher’s g-test are shown in the logarithmic scale (X-axis).

**
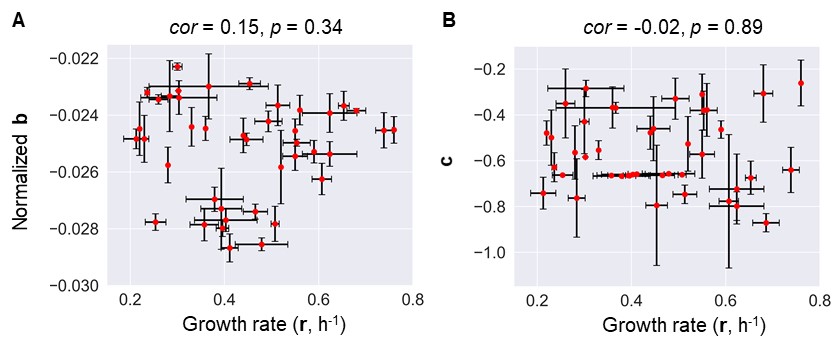
**

**Figure S3 Scatter plots of the periodic parameters (b and c) and the growth rate.** The theoretical meaning and the calculation of these parameters were described in the main text, as well as shown in Fig. 2. The standard errors of biological replicates are indicated. The correlation coefficient and its significance are indicated.

**
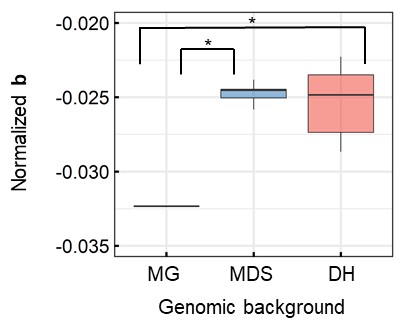
**

**Figure S4 Boxplot of the periodic parameter b.** The normalized parameter **b** was classified in accordance with the genomes. MG, MDS, and DH indicate the wild-type genome of MG1655, the reduced genome of MDS42 and its derivatives, and the genomes of an assortment of genetically engineered DH1 strains, respectively. The details are summarized in Table S1. Asterisk indicates *p* < 0.05.

**
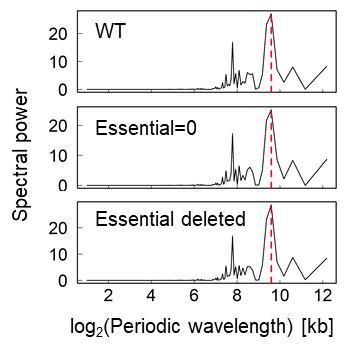
**

**Figure S5 Contribution of essential genes to chromosomal periodicity.** The distribution of the Fourier-transformed periodic wavelength at a logarithmic scale is shown. The MG1655 transcriptomes under normal conditions were subjected to analysis. The upper, middle and bottom panels represent the transcriptomes of all genes in the wild-type genome, which were determined by replacing the expression data of the essential genes with zero and removing the expression data of the essential genes. The most significant spectral power is indicated with a red broken line.

**
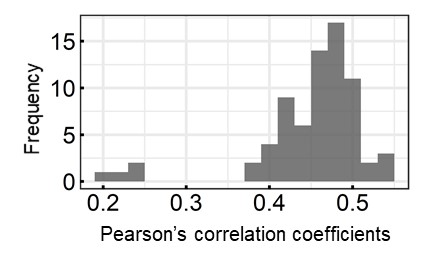
**

**Figure S6 Histogram of the correlation coefficients between GyrA binding activity and gene expression levels.** A total of 213 transcriptomes were all subjected to the correlation analysis as performed in Fig. 3B. Note that the analysis was based on the hypothesis of that GyrA binding activity remained unchanged in all genomes and growth conditions. The analysis resulted in 213 values of correlation coefficients, which are shown in the histogram.

**
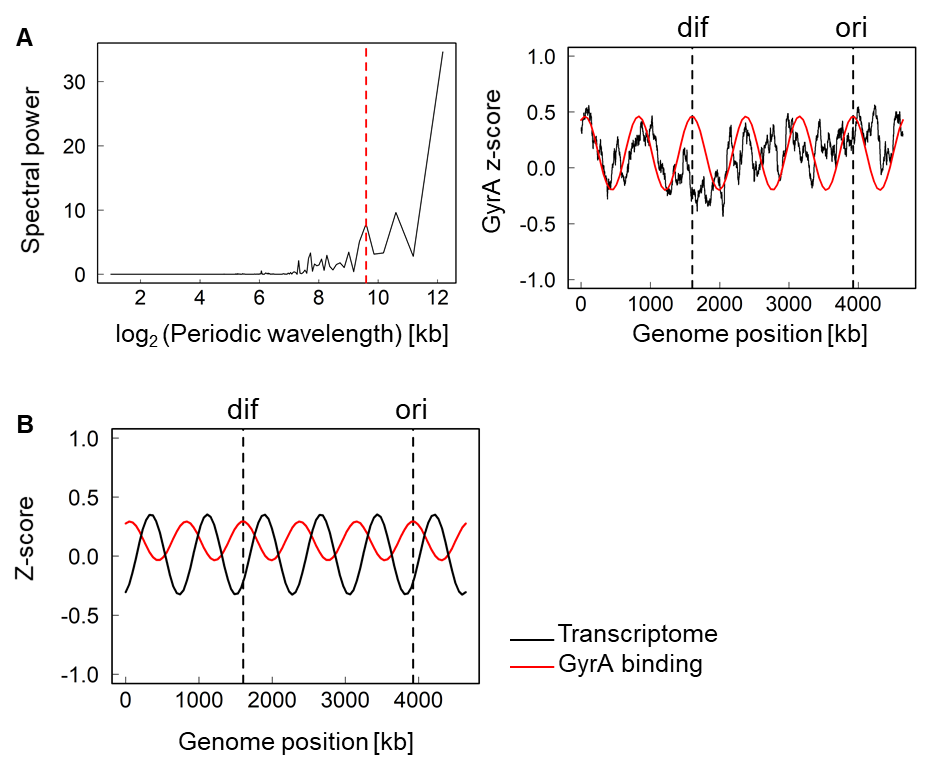
**

**Figure S7 Chromosomal periodicity of GyrA binding activity. A.** Fourier transform of the GyrA binding activity. The left and right panels represent the distributions of the Fourier-transformed periodic wavelengths at the logarithmic scale and the estimated chromosomal periodicity of the GyrA binding activity, respectively. The broken lines and solid curves in red indicate the spectral power corresponding to the six periods, as estimated by the Fourier transform and the corresponding fitted period of the GyrA binding activity, respectively. The GyrA binding activity (Chip-seq values) for every 1 kb sliding window and 100 kb smoothing are shown. *Ori* and *dif* are indicated by the broken lines in black. **C.** Chromosomal periodicity of the transcriptome and GyrA binding activity. As the transcriptomes showed a conserved chromosomal periodicity of six periods, the spectral power of GyrA binding activity corresponding to the six periods was used for the comparison. Red and black curves indicate the periodicity of the GyrA binding activity and the transcriptome, respectively. Both were calculated using a 1 kb sliding window and are shown as a 100 kb moving average. *Ori* and *dif* are indicated by the broken lines in black.

**
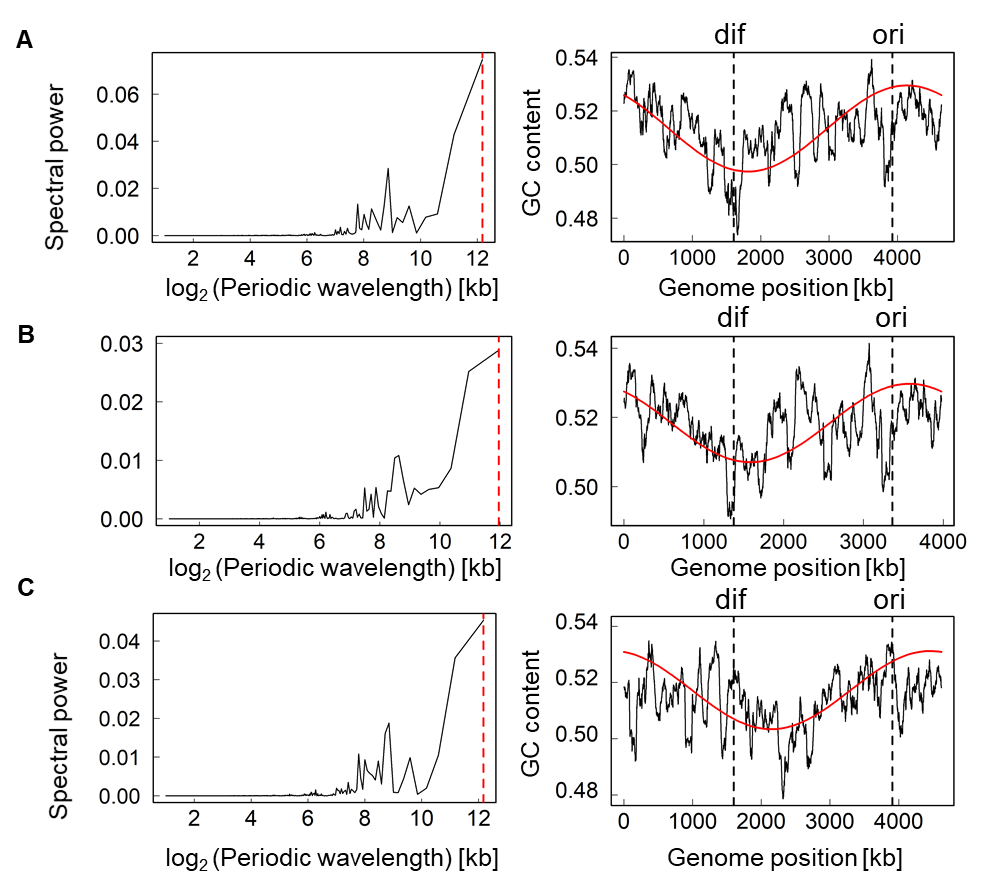
**

**Figure S8 Chromosomal periodicity of GC content.** Fourier transform of the GC contents per gene of MG1655 (**A**), MDS42 (**B**), and DH1 (**C**) were performed. The left and right panels represent the distributions of the Fourier-transformed periodic wavelengths at the logarithmic scale and the estimated chromosomal periodicity of the GC content, respectively. The broken lines and solid curves in red indicate the spectral power corresponding to the single period, as estimated by the Fourier transform, and the corresponding fitted period of the GC contents, respectively. The GC content for every 1 kb sliding window and 100 kb smoothing are shown. *Ori* and *dif* are indicated by the broken lines in black.
